# Longitudinal association of the anti-inflammatory serum marker GDF-15 with serum IgA and IgG in apparently healthy children

**DOI:** 10.1038/s41598-021-97386-1

**Published:** 2021-09-14

**Authors:** Gemma Carreras-Badosa, Ariadna Gómez-Vilarrubla, Berta Mas-Parés, Silvia Xargay-Torrent, Anna Prats-Puig, Elsa Puerto-Carranza, Francis de Zegher, Lourdes Ibáñez, Judit Bassols, Abel López-Bermejo

**Affiliations:** 1 Pediatric Endocrinology Group, Girona Biomedical Research Institute, Av. França s/n, 17007 Girona, Spain; 2 Maternal-Fetal Metabolic Group, Girona Biomedical Research Institute, Girona, Spain; 3 University School of Health and Sport (EUSES), University of Girona, Girona, Spain; 4 Dr. Josep Trueta Hospital, Girona, Spain; 5 Department of Development and Regeneration, University of Leuven, Leuven, Belgium; 6 Sant Joan de Déu Children’s Hospital Pediatric Institute, University of Barcelona, Barcelona, Spain; 7 CIBERDEM, Instituto de Salud Carlos III, Madrid, Spain; 8 Department of Medical Sciences, University of Girona, Girona, Spain

**Keywords:** Adaptive immunity, Endocrine system and metabolic diseases

## Abstract

Both the innate and adaptive immune responses are deregulated in individuals with obesity and are key drivers of its associated metabolic alterations. Although the anti-inflammatory growth differentiation factor 15 (GDF-15) is a candidate protein against obesity, its mechanisms regulating the immune responses are not fully cleared. We examined whether GDF-15 was related to serum immunoglobulins in a children’s cohort assessed longitudinally during childhood. Results showed that circulating GDF-15 positively associated with IgA (p < 0.002) and IgG (p < 0.001) levels and the IgA*IgG product (p < 0.001) in apparently healthy children at both baseline (age 9) and follow-up (age 13). The associations were readily observed in heavier children (those with BMI-SDS above the median) as well as in children with higher renal fat accumulation (those with renal fat-to-height ratio above the median) and remained significant after correcting for possible confounding variables. Serum GDF-15 levels accounted for up to 16% of the variance of follow-up IgG levels and up to 14% of the variance of follow-up IgA*IgG product. The longitudinal associations of the anti-inflammatory GDF-15 with IgA, IgG and the IgA*IgG product in children with higher BMI or higher renal fat accumulation suggest a role of GDF-15 in human obesity through the regulation of the immune adaptive system.

## Introduction

Obesity and its associated metabolic alterations have reached epidemic proportions, also in childhood^1^. A crucial driver of this process is the chronic low-grade inflammation observed in individuals with obesity. Chronic inflammation is known to have the active participation of innate immune system, including T cell activation and production of immunoglobulins by B lymphocytes infiltrating the visceral adipose tissue^2,3^. Growth differentiation factor 15 (GDF-15) is a candidate protein in the fight against obesity because of its anti-inflammatory, anorexigenic and lipolytic properties^4^. Despite the fact that GDF-15 is recognized as a key circulating anti-inflammatory factor, scarce publications in obese children have been reported^5^ and its mechanisms regulating immune responses are not fully elucidated. IgA and IgG have been related to a poorer metabolic profile in obese children^6^, and. higher concentrations of IgA and IgG have been reported in obese adults^7^ and children^8^, suggesting that there is a relationship between the adaptive immune system and adipose tissue function. We hypothesized that GDF-15 would be associated with components of the immune system in children with obesity.

The aim of this study was to examine whether GDF-15 was related to serum levels of IgA and IgG in a cohort of apparently healthy children assessed longitudinally during childhood both at baseline and at follow-up. Analyses were also performed in groups thereof with higher body mass index (BMI) or renal fat.

## Methods

The study population consisted of 204 Caucasian children (101 girls and 103 boys) assessed longitudinally at baseline (8.5 ± 1.8 years) and at follow-up (13.0 ± 1.9 years). Subjects were consecutively recruited among those seen in a primary care setting in Girona, a region in North-eastern Spain, and included in a previously reported children’s cohort^9,10^. Briefly, inclusion criteria were (1) age between 6 and 10 years; (2) no pubertal development, as judged by a specifically trained nurse using Tanner criteria (breast stage I; testicular volume bilaterally < 4 ml). Exclusion criteria were (1) major congenital anomalies; (2) abnormal blood count, abnormal liver, kidney or thyroid functions; (3) evidence of chronic illness or prolonged use of medication; (4) acute illness or use of medication in the month preceding potential enrolment. The study protocol was approved by the Ethics Review Committee of the Institutional Review Board of Dr. Josep Trueta Hospital and was performed in accordance with their code of ethics, guidelines and regulations. Informed written consent was obtained from the parents.

Clinical examination followed by venous blood sampling in the fasting state was performed in the morning as previously reported^9,10^. Briefly, weight and height were measured with a calibrated scale and a Harpenden stadiometer, respectively. Body mass index (BMI) was calculated as weight divided by the square of height in meters. Age-adjusted and sex-adjusted standard deviation scores (SDS) for BMI were calculated using regional normative data. Renal fat thickness was assessed by high-resolution ultrasonography using a linear 12-MHz transducer (MyLabTM25, Esaote). Averages of three to five measurements were used in the study. All measurements were performed by the same observer who was unaware of the clinical and laboratory characteristics of the subjects. Intra-subject coefficient of variation for ultrasound measurements was less than 6%. Blood pressure was measured in the supine position on the right arm after a 10-min rest using an electronic sphygmomanometer with cuff size appropriate for children’s arm circumference.

All serum samples were obtained between 8:00 and 9:00 AM under fasting conditions. Fasting serum immunoreactive insulin was assayed as described previously^9^. Total IgG, IgA and IgM were measured by a commercial nephelometric immunoassay (Immage Immunochemistry Systems; Beckman Coulter). The intra-assay CVs were 2.0–2.6%, 2.5–2.9% and 2.4–3.2%, respectively. GDF-15 was measured by an ELISA (Human GDF-15 DuoSet ELISA, R&D Systems). The sensitivity was 7 pg/ml and intra-assay CVs were less than 8%. Serum samples were kept frozen at -80 °C until assay. Follow-up data were obtained 4 years after the baseline visit and the same anthropometric, clinical and laboratory variables (except for GDF-15) were assessed following the same methodology.

Statistical analyses were performed using SPSS version 22.0 (SPSS Inc.). Results are expressed as mean ± standard deviation (SD). Logarithmic transformation was used to obtain normally distributed values for GDF-15 and immunoglobulins. Differences across groups defined by the median of BMI-SDS or the median of renal fat-to-height ratio were examined by independent T-test (continuous data) and by Chi square (categorical data). The relation between variables was analyzed by Pearson bivariate correlations followed by multivariate linear regression analyses. The enter method was used for computing the independent variables and the step-wise method was used for additionally computing individual R^2^ values. Significance level was set at p < 0.05.

## Results

The total studied population comprised 204 apparently healthy children with a mean BMI-SDS of 0.62 ± 1.40. Circulating baseline GDF-15 levels ranged from 35 to 221 pg/ml (Table 1). Subjects were split into subgroups according to the median value of baseline BMI-SDS (Supplementary Table S1) and according to the median value of baseline renal fat-to-height ratio (as a proxy of visceral fat accumulation; Table 1). Differences between subgroups according to renal fat-to-height ratio were seen in almost all anthropometric parameters being analyzed such as weight, height, BMI and renal fat (Table 1).

**Table 1:** Descriptive analysis of the studied parameters in all the children and in subgroups thereof defined by the median value of the renal fat-to-height ratio.

	All subjects	Below renal fat-to-height ratio median	Above renal fat-to-height ratio median
	N = 204	N = 102	N = 102
**Baseline**			
Female/male sex (n)	101/103	50/52	51/51
Overweight (n)	62	19	43***
Age (years)	8.5 ± 1.8	8.4 ± 1.7	8.6 ± 1.8
Weight (kg)	37.8 ± 14.7	34.9 ± 12.7	40.5 ± 16.2**
Weight-SDS (z-score)	0.82 ± 1.45	0.51 ± 1.35	1.14 ± 1.49**
Height (cm)	135.4 ± 12.9	134.9 ± 12.7	135.7 ± 13.3
Height-SDS (z-score)	0.69 ± 1.13	0.69 ± 1.20	0.67 ± 1.06
BMI (kg/m2)	19.9 ± 4.7	18.6 ± 3.8	21.1 ± 5.1***
BMI-SDS (z-score)	0.62 ± 1.40	0.23 ± 1.21	1.00 ± 1.47***
Renal fat (cm)	0.20 ± 0.04	0.17 ± 0.03	0.24 ± 0.03***
Renal fat-to-height ratio (cm/m)	0.15 ± 0.03	0.12 ± 0.02	0.18 ± 0.02***
SBP (mmHg)	108 ± 11	107 ± 10	109 ± 11
DBP (mmHg)	61 ± 7	61 ± 7	61 ± 7
Insulin (μlU/mL)	5.6 ± 5.6	5.1 ± 4.1	6.1 ± 5.4
IgM (mg/dL)	100 ± 42	103 ± 40	98 ± 42
IgA (mg/dL)	108 ± 52	106 ± 43	113 ± 58
IgG (mg/dL)	982 ± 220	961 ± 212	994 ± 216
IgA*IgG product × 10^3^	110 ± 71	105 ± 57	118 ± 83
GDF-15 (pg/mL)	99.3 ± 28.0	101.6 ± 29.2	97.3 ± 26.9
**Follow-up**			
Overweight (n)	61	22	39*
Age (years)	13.0 ± 1.9	12.9 ± 1.8	13.0 ± 1.9
Weight (kg)	59.1 ± 19.9	55.4 ± 17.7	62.7 ± 21.4**
Weight-SDS (z-score)	0.84 ± 1.51	0.50 ± 1.29	1.19 ± 1.64**
Height (cm)	158.6 ± 12.1	158.2 ± 12.3	158.6 ± 12.1
Height-SDS (z-score)	0.42 ± 1.03	0.40 ± 0.94	0.44 ± 1.11
BMI (kg/m2)	23.2 ± 6.0	21.7 ± 5.0	24.6 ± 6.6**
BMI-SDS (z-score)	0.76 ± 1.58	0.36 ± 1.33	1.16 ± 1.71***
Renal fat (cm)	0.12 ± 0.04	0.11 ± 0.03	0.13 ± 0.05**
Renal fat-to-height ratio (cm/m)	0.07 ± 0.02	0.07 ± 0.02	0.08 ± 0.03**
SBP (mmHg)	115 ± 12	113 ± 11	117 ± 13*
DBP (mmHg)	62 ± 8	62 ± 7	62 ± 8
Insulin (μlU/mL)	11.5 ± 6.9	10.4 ± 6.1	12.6 ± 5.6*
IgM (mg/dL)	118 ± 49	123 ± 49	113 ± 49
IgA (mg/dL)	135 ± 59	134 ± 54	138 ± 63
IgG (mg/dL)	1006 ± 223	983 ± 206	1019 ± 229
IgA*IgG product × 10^3^	140 ± 82	136 ± 68	148 ± 94

Data are shown as mean ± standard deviation (SD) values.

*SDS* standard deviation score, *BMI* body mass index, *SBP* systolic blood pressure, *DBP* diastolic blood pressure, *Ig* immunoglobulin, *GDF-15* growth differentiation factor 15.

Independent t-test, *P < 0.05, **P < 0.01, ***P < 0.001 as compared to lower renal fat-to-height ratio median group.

**Figure 1:**
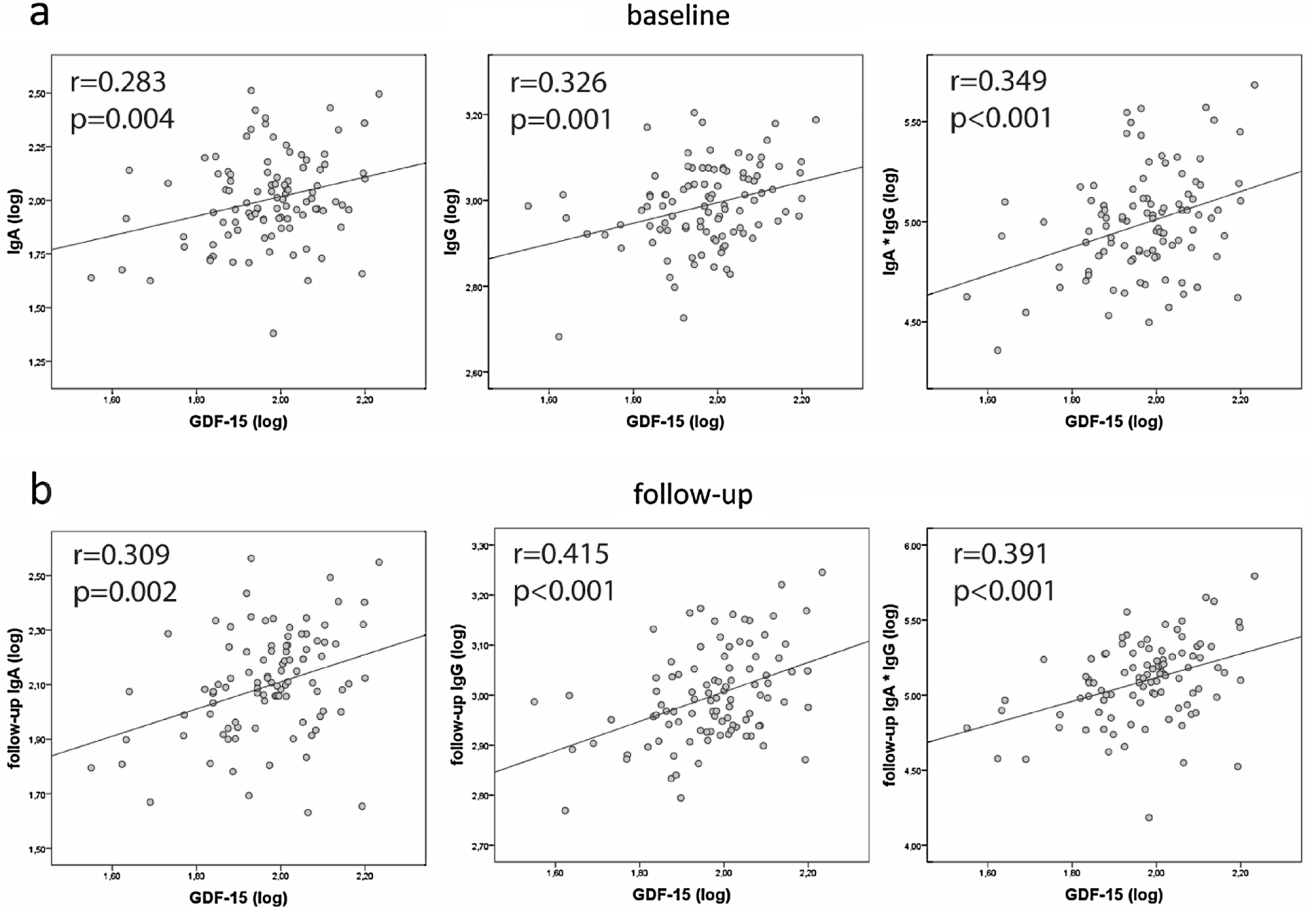
Correlation graphs between circulating GDF-15 and the studied IgA, IgG and IgA*IgG product in children with higher renal fat-to-height ratio (above the median; N = 102). (**a**) At baseline: 9 years of age and (**b**) at follow-up: 13 years of age.

Bivariate correlations showed that GDF-15 was positively associated at baseline with IgG levels and with the IgA*IgG product in all studied subjects (mean age 9 years; all p < 0.05; data not shown). Moreover, when analyzing the subgroups defined by the median of BMI-SDS or the median of renal fat-to-height ratio, children with higher BMI-SDS (data not shown) and especially those with higher renal fat-to-height showed positive correlations between circulating GDF-15 and IgA (p = 0.004), IgG (p = 0.001) levels and the IgA*IgG product (p < 0.001; Fig. 1a).

Follow-up data at a mean age of 13 years showed that baseline GDF-15 was also positively associated with follow-up IgA and IgG levels as well as the IgA*IgG product, when analyzing all subjects or those children with BMI-SDS above the median (all p < 0.05; data not shown), but especially when analyzing those children with renal fat-to-height ratio above the median (all p < 0.002; Fig. 1b).

Finally, multivariate regression analyses assessed on the previously observed correlations showed that circulating GDF-15 remained independently associated with IgA, IgG and the IgA*IgG product in children with BMI-SDS or renal fat-to-height ratio above the median at baseline, after correcting for possible confounding variables such as age, sex and BMI (Supplementary Table S2). In these models, baseline GDF-15 levels accounted for up to 10% of the variance of IgG levels and up to 6% of the variance of the IgA*IgG product.

At follow-up, baseline GDF-15 remained also independently associated with follow-up IgA, IgG and the IgA*IgG product in children with BMI-SDS or renal fat-to-height ratio above the median, after correcting for the same confounding variables (Supplementary Table S2). In these models, baseline GDF-15 explained up to 16% of the variance of follow-up IgG levels and up to 14% of the variance of the follow-up IgA*IgG product.

## Discussion

The main result of the study is that GDF-15 associates positively and longitudinally with IgA, IgG and IgA*IgG product in children with higher BMI or higher renal fat accumulation.

As previously reported by our group and others, IgA and IgG are upregulated in obesity^7,8^ and related to a poorer metabolic profile in children^6^. Hence, immunoglobulins such as IgA and IgG seem to be closely related to the altered metabolic status in obesity. Although immunoglobulins present functional diversification, IgG is often described as pathogenic^11^ and IgA has been demonstrated to be an inducer of inflammation^12^.

B lymphocytes, which secrete IgA and IgG antibodies, have been shown to be among the first cells to be recruited into visceral adipose tissue, followed by recruitment of T cells^3^, which in turn could dictate the extent of the local inflammatory response through macrophage modulation and secretion of pro-inflammatory cytokines^13^. GDF-15 is known to be secreted as an adipokine^14^, and similarly to other adipokines may regulate the innate and adaptive immune response^15^. Along the same line, GDF-15 was shown to inhibit T cell stimulation and reduce dendritic cell maturation markers in dendritic cell cultures^16^. Others have shown that GDF15 is up-regulated as a physiological counter-regulatory mechanism in disorders associated with cell stress^17^. Finally, a relationship between GDF-15 and immunoglobulins has been also found, for example, in subjects with IgG4-related disease^18^ or patients with IgA nephropathy^19^.

Taking into account our results and the previous literature discussed above it is plausible to speculate that the anti-inflammatory GDF-15 could regulate the adaptive immunity by means of counteracting the pro-inflammatory effects of IgA and IgG and thus reducing the obesity-related low-grade inflammation state.

We acknowledge some limitations of our study such as not observing any direct associations between GDF-15 and obesity parameters, for instance, BMI or renal fat in our studied children. One explanation might be that in our study, children were overall healthy. It has been suggested that a threshold degree of obesity is necessary to elicit an adaptive immune system response in adipose tissue^20^. Accordingly, GDF-15 correlations with IgA and IgG were significant in heavier children (those with BMI-SDS above the median) and in children with higher accumulation of renal fat (those with renal fat-to-height above the median), which give support to a possible role of GDF-15 in obesity-triggered inflammation. Among the strengths of our study is the longitudinal independent associations between GFD-15, IgA, IgG and the IgA*IgG, suggesting a causal role of GFD-15 in the regulation of adaptive immunity.

In conclusion, serum GDF-15 associates positively and longitudinally with IgA, IgG and the IgA*IgG product in children with higher BMI or higher renal fat accumulation. Our results suggest that GDF-15 could exert its known anti-inflammatory activity by regulating at least in part the adaptive immunity, especially in heavier subjects or in those with more visceral fat accumulation. Further research studies may help disclose whether GDF-15 treatment can limit the pro-inflammatory effects of IgA and IgG in obesity.

## Supplementary Information

Supplementary Information.

## Data Availability

The datasets analyzed during the current study are available from the corresponding author on reasonable request.
