# Supplementary material for: Longitudinal association of the anti-inflammatory serum marker GDF-15 with serum IgA and IgG in apparently healthy children
Source: Sci Rep. 2021 Sep 14;11:18215. doi: 10.1038/s41598-021-97386-1 (PMC8440501; doi:10.1038/s41598-021-97386-1)
Supplement: Supplementary file 1 — Supplementary Information. [file 41598_2021_97386_MOESM1_ESM.docx]

**Longitudinal association of the anti-inflammatory serum marker GDF-15 with serum IgA and IgG in apparently healthy children**

Gemma Carreras-Badosa^1^, Ariadna Gomez-Vilarrubla^2^, Berta Mas-Parés^1^, Silvia Xargay-Torrent^1^, Anna Prats-Puig^3^, Elsa Puerto-Carranza^1, 4^, Francis de Zegher^5^, Lourdes Ibañez^6, 7^, Judit Bassols^2^, Abel López-Bermejo* ^1, 4, 8^

^1^ Pediatric Endocrinology Group, Girona Biomedical Research Institute, Girona, Spain;

^2^ Maternal-Fetal Metabolic Group, Girona Biomedical Research Institute, Girona, Spain;

^3^ University School of Health and Sport (EUSES), University of Girona, Girona, Spain;

^4^ Pediatrics, Dr. Josep Trueta Hospital, Girona, Spain;

^5^ Department of Development & Regeneration, University of Leuven, Leuven, Belgium;

^6^ Sant Joan de Déu Children’s Hospital Pediatric Institute, University of Barcelona, Barcelona, Spain;

^7^ CIBERDEM, Instituto de Salud Carlos III, Madrid, Spain;

^8^ Department of Medical Sciences, University of Girona, Girona, Spain.

**CORRESPONDING AUTHOR**

Abel López-Bermejo, MD

Girona Institute for Biomedical Research

Av. França s/n

Girona-17007. Spain

Tel: +34-972-940200. Ext. 2810

Fax: +34-972-940270

Email: [alopezbermejo@idibgi.org](mailto:alopezbermejo@idibgi.org)

**RUNNING TITLE:** GDF-15 associates with IgA and IgG in children

**KEY WORDS:** GDF-15, obesity, immunoglobulins, adaptive immunity

**Supplementary Table S1.** Descriptive analysis of the studied parameters in all the children and in subgroups thereof defined by the median value of BMI-SDS.

|  | **All subjects** | **Below BMI-SDS median** | **Above BMI-SDS median** |
| --- | --- | --- | --- |
|  | **N=204** | **N=102** | **N=102** |
| **Baseline** | | | |
| Female/male sex (n) | 101/103 | 53/49 | 48/54 |
| Overweight (n) | 62 | 0 | 62*** |
| Age (years) | 8.5 ± 1.8 | 8.0 ± 1.7 | 9.1 ± 1.7*** |
| Weight (kg) | 37.8 ± 14.7 | 27.2 ± 7.2 | 48.4 ± 12.6*** |
| Weight-SDS (z-score) | 0.82 ± 1.45 | -0.37 ± 0.71 | 2.02 ± 0.93*** |
| Height (cm) | 135.4 ± 12.9 | 129.4 ± 11.4 | 141.4 ± 11.5*** |
| Height-SDS (z-score) | 0.69 ± 1.13 | 0.21 ± 1.10 | 1.17 ± 0.95*** |
| BMI (kg/m2) | 19.9 ± 4.7 | 16.0 ± 1.9 | 23.8 ± 3.2*** |
| BMI-SDS (z-score) | 0.62 ± 1.40 | -0.57 ± 0.61 | 1.80 ± 0.85*** |
| Renal fat (cm) | 0.20 ± 0.04 | 0.19 ± 0.04 | 0.22 ± 0.05*** |
| Renal fat-to-height ratio (cm/m) | 0.15 ± 0.03 | 0.14 ± 0.03 | 0.16 ± 0.03 |
| SBP (mmHg) | 108 ± 11 | 104 ± 9 | 111 ± 11*** |
| DBP (mmHg) | 61 ± 7 | 60 ± 6 | 63 ± 8** |
| Insulin (μlU/mL) | 5.6 ± 5.6 | 2.8 ± 2.8 | 8.3 ± 6.6*** |
| IgM (mg/dL) | 100 ± 42 | 106 ± 41 | 95 ± 42 |
| IgA (mg/dL) | 108 ± 52 | 97 ± 44 | 119 ± 57** |
| IgG (mg/dL) | 982 ± 220 | 968 ± 237 | 997 ± 202 |
| IgA*IgG product x10^3^ | 110 ± 71 | 96 ± 57 | 124 ± 81*** |
| GDF-15 (pg/mL) | 99.3 ± 28.0 | 99.0 ± 28.5 | 99.6 ± 27.7 |
| **Follow-up** | | | |
| Overweight (n) | 61 | 0 | 61*** |
| Age (years) | 13.0 ± 1.9 | 12.3 ± 1.8 | 13.6 ± 1.8*** |
| Weight (kg) | 59.1 ± 19.9 | 44.2 ± 11.2 | 74.0 ± 15.0*** |
| Weight-SDS (z-score) | 0.84 ± 1.51 | -0.31 ± 0.75 | 1.98 ± 1.18*** |
| Height (cm) | 158.6 ± 12.1 | 153.7 ± 12.4 | 163.4 ± 9.8*** |
| Height-SDS (z-score) | 0.42 ± 1.03 | 0.17 ± 1.10 | 0.67 ± 0.90*** |
| BMI (kg/m2) | 23.2 ± 6.0 | 18.4 ± 2.5 | 27.9 ± 4.6*** |
| BMI-SDS (z-score) | 0.76 ± 1.58 | -0.46 ± 0.65 | 1.97 ± 1.27*** |
| Renal fat (cm) | 0.12 ± 0.04 | 0.10 ± 0.02 | 0.15 ± 0.04*** |
| Renal fat-to-height ratio (cm/m) | 0.07 ± 0.02 | 0.07 ± 0.01 | 0.09 ± 0.02*** |
| SBP (mmHg) | 115 ± 12 | 109 ± 11 | 121 ± 11*** |
| DBP (mmHg) | 62 ± 8 | 60 ± 7 | 64 ± 8*** |
| Insulin (μlU/mL) | 11.5 ± 6.9 | 8.6 ± 4.0 | 14.5 ± 7.9*** |
| IgM (mg/dL) | 118 ± 49 | 122 ± 49 | 114 ± 49 |
| IgA (mg/dL) | 135 ± 59 | 119 ± 53 | 151 ± 62*** |
| IgG (mg/dL) | 1006 ± 223 | 978 ± 220 | 1033 ± 224 |
| IgA*IgG product x10^3^ | 140 ± 82 | 119 ± 68 | 161 ± 90*** |

Data are shown as mean ± standard deviation (SD) values.

SDS: standard deviation score, BMI: body mass index, SBP: systolic blood pressure, DBP: diastolic blood pressure, TG: triglycerides, Ig: immunoglobulin, GDF-15: growth differentiation factor 15.

Independent t-test, *P < 0.05, **P < 0.01, ***P < 0.001 as compared to lower BMI-SDS median group.

**Supplementary Table S2.** Multivariate analyses between circulating GDF-15 and the studied parameters in children at 9 and follow-up at 13 years of age in:

1. Children with BMI-SDS above the median (N=102).

|  | **Baseline** | | | | | | | **Follow-up** | | | | | | |
| --- | --- | --- | --- | --- | --- | --- | --- | --- | --- | --- | --- | --- | --- | --- |
|  | **IgA** | | | **IgG** | | **IgA*IgG product** | | **IgA** | | **IgG** | | | **IgA*IgG product** | |
|  | **β** | **P value** | **β** | | **P value** | **β** | **P value** | **β** | **P value** | | **β** | **P value** | **β** | **P value** |
| Baseline GDF-15 | 0.184 | 0.049 | 0.223 | | 0.017 | 0.246 | 0.007 | 0.253 | 0.010 | | 0.238 | 0.018 | 0.341 | < 0.001 |
| Sex (female/male) | -0.134 | ns | -0.302 | | 0.001 | -0.207 | 0.021 | -0.074 | ns | | -0.200 | 0.043 | -0.153 | ns |
| Baseline age | 0.226 | 0.046 | 0.142 | | ns | 0.184 | ns | 0.131 | ns | | 0.102 | ns | 0.099 | ns |
| Baseline BMI | 0.198 | ns | 0.168 | | ns | 0.225 | 0.040 | 0.181 | ns | | 0.004 | ns | 0.188 | ns |
| ***Model R^2^*** | *0.173* | | | *0.189* | | *0.218* | | *0.114* | | *0.073* | | | *0.175* | |
| ***GDF-15 R^2*^*** | *0.012* | | | *0.033* | | *0.049* | | *0.043* | | *0.048* | | | *0.105* | |

**Step-wise method.*

1. Children with renal fat-to-height ratio above the median (N=102).

|  | **Baseline** | | | | | | | **Follow-up** | | | | | | |
| --- | --- | --- | --- | --- | --- | --- | --- | --- | --- | --- | --- | --- | --- | --- |
|  | **IgA** | | | **IgG** | | **IgA*IgG product** | | **IgA** | | **IgG** | | | **IgA*IgG product** | |
|  | **β** | **P value** | **β** | | **P value** | **β** | **P value** | **β** | **P value** | | **β** | **P value** | **β** | **P value** |
| Baseline GDF-15 | 0.192 | 0.036 | 0.283 | | 0.003 | 0.259 | 0.003 | 0.241 | 0.013 | | 0.404 | < 0.001 | 0.333 | < 0.001 |
| Sex (female/male) | -0.189 | 0.035 | -0.296 | | 0.002 | -0.261 | 0.003 | -0.162 | ns | | -0.244 | 0.009 | -0.214 | 0.019 |
| Baseline age | 0.396 | 0.001 | 0.207 | | ns | 0.395 | < 0.001 | 0.186 | ns | | 0.044 | ns | 0.164 | ns |
| Baseline BMI | 0.019 | ns | -0.026 | | ns | 0.006 | ns | 0.163 | ns | | -0.001 | ns | 0.129 | ns |
| ***Model R^2^*** | *0.235* | | | *0.189* | | *0.302* | | *0.178* | | *0.199* | | | *0.229* | |
| ***GDF-15 R^2*^*** | *0.028* | | | *0.097* | | *0.058* | | *0.051* | | *0.164* | | | *0.144* | |

**Step-wise method.*
